# Current Understanding of the Etiology, Symptomatology, and Treatment Options in Premature Ovarian Insufficiency (POI)

**DOI:** 10.3389/fendo.2021.626924

**Published:** 2021-02-25

**Authors:** Bunpei Ishizuka

**Affiliations:** ^1^Rose Ladies Clinic, Tokyo, Japan; ^2^Department of Obstetrics and Gynecology, St. Marianna University School of Medicine, Kanagawa, Japan

**Keywords:** premature ovarian insufficiency (POI), hormone replacement therapy (HRT), chromosomal abnormality, autoimmunity, genetics, vasomotor symptoms, cardiovascular disease, ovulation induction

## Abstract

Premature ovarian insufficiency (POI) occurs in at least 1% of all women and causes life-long health problems and psychological stress. Infertility caused by POI used to be considered absolute, with infertility treatment having little or no value. Generally, it has been thought that medicine can provide little service to these patients. The etiology of POI has been found to be genetic, chromosomal, and autoimmune. In addition, the increasing numbers of cancer survivors are candidates for iatrogenic POI, along with patients who have undergone ovarian surgery, especially laparoscopic surgery. Over 50 genes are known to be causally related to POI, and the disease course of some cases has been clarified, but in most cases, the genetic background remains unexplained, suggesting that more genes associated with the etiology of POI need to be discovered. Thus, in most cases, the genetic background of POI has not been clarified. Monosomy X is well known to manifest as Turner’s syndrome and is associated with primary amenorrhea, but recent studies have shown that some women with numerical abnormalities of the X chromosome can have spontaneous menstruation up to their twenties and thirties, and some even conceive. Hormone replacement therapy (HRT) is recommended for women with POI from many perspectives. It alleviates vasomotor and genitourinary symptoms and prevents bone loss and cardiovascular disease. POI has been reported to reduce quality of life and life expectancy, and HRT may help improve both. Most of the problems that may occur with HRT in postmenopausal women do not apply to women with POI; thus, in POI, HRT should be considered physiological replacement of estrogen (+progesterone). This review describes some new approaches to infertility treatment in POI patients that may lead to new treatments for POI, along with the development of more sensitive markers of secondary/preantral follicles and genetic diagnosis.

## Introduction

The diagnosis of premature ovarian insufficiency (POI) is a serious event for a woman. It is a condition with medical, psychological, and reproductive implications. It causes menstrual disturbances with infertility, as well as various health problems, mainly due to the estrogen deficiency, throughout women’s lives. POI is associated, in the long term, with an increased risk of cardiovascular disorders and osteoporosis and with some degree of cognitive deterioration. In addition, POI is reported to be associated with earlier mortality (1).

After a diagnosis of POI, fertility treatment has been considered to be of no or little value.

This review summarizes the current issues related to etiological factors, symptomatology, and treatment options in POI.

## Definition, Diagnosis, and Prevalence

POI is a state in which ovarian function decreases irreversibly beyond the extent of the normal range for the women’s age. It can manifest as primary or secondary amenorrhea. In cases with secondary amenorrhea, menstrual cycle changes may precede the amenorrhea (2).

Follicle stimulating hormone (FSH) levels have been mainly used in making the diagnosis of POI, but precise cut-off levels have not been determined. Initially, a number of papers used FSH levels >40, 50, or 20 mIU/ml as the criteria based on older reports, but some patients with POI sometimes show FSH levels lower than these cut-off levels. Thus, diagnostic accuracy for POI is low, though early diagnosis is mandatory in the treatment of all phases of problems associated with POI. The diagnosis of POI should be based on the presence of a menstrual disturbance with biochemical confirmation.

Thus, the most appropriate diagnostic criteria proposed so far are those of the European Society of Human Reproduction and Embryology (ESHRE) POI guideline development group’s guideline, which are as follows: oligo/amenorrhea for at least 4 months and elevated FSH levels >25 mIU/ml on two occasions >4 weeks apart.

The prevalence of POI was originally reported by Caulum et al. as being 1% of all women in a longitudinal, cohort study involving 1,858 women born between 1928 and 1932 (3). Luborsky et al. reported that the incidence of POI was 1.1% in 2003 in a cross-sectional study. They also reported that the prevalence of POI in the USA was higher in African-American and Hispanic women than in Caucasian women and lower in Asian American women (4). A recent national registry study in Sweden reported that the incidence was 1.8% (5). On the other hand, a meta-analysis of the global prevalence of POI found a rate of 3.7% (6).

Thus, the prevalence of POI may have ethnic and/or regional differences and change with time due to lifestyle and/or environmental factors that still need to be further investigated.

## Etiological Factors

Whereas the cause of POI is unknown in many cases, a familial trait has long been recognized. The prevalence of familial POI has been reported to be 4 to 31% in various studies (7–10).

Most of the genetic factors related to the etiology of POI are unknown. The etiological factors known to cause familial POI are chromosomal abnormalities and some gene mutations.

### Chromosomal Abnormalities

An analysis of 688 spontaneous POI cases seen at the St. Marianna University School of Medicine and the Rose Ladies Clinic found karyotype abnormalities in 88 (12.8%) of the cases (**Table 1**) (11). Most of them were abnormalities of the X chromosome, although a few autosomal abnormalities were observed. Both numerical abnormalities and terminal deletion of the X chromosome can cause POI.

**Table 1:** Possible etiological factors in 827 POI patients diagnosed at St. Marianna University School of Medicine and the Rose Ladies Clinic (11).

● Idiopathic	n = 688 (83.2%)
• Normal karyotype	n = 600 (87.2%)
Autoantibody-positive	n = 270 (45.0%)
Autoantibody-negative	n = 330 (55.0%)
• Chromosomal abnormality	n = 88 (12.8%)
● Iatrogenic	n = 131 (15.8%)
• Post-chemo and/or radio-therapy	n = 47 (35.9%)
• Post-ovarian surgery	n = 84 (64.1%)
● Post-inflammation	n = 8 (1.0%)
Total	n = 827

Patients with monosomy X (45,XO) mostly show primary amenorrhea and signs of Turner’s syndrome, but cases with mosaicism with 46,XX show the typical course of POI, and the age at onset of amenorrhea tends to be older as the 45,XO ratio decreases (**Table 2**) (11). There are a few cases of monosomy X with primary amenorrhea who develop spontaneous pregnancy (12). In patients with primary amenorrhea, the rate of an abnormal karyotype is higher than in those with secondary amenorrhea (13).

**Table 2:** Karyotypes and ages at onset of amenorrhea in 42 representative POI cases with chromosomal abnormalities (11).

No.	Age at the onset of amenorrhea (y)	Karyotypes	Turner Stigmata	Follicle Growth	Ovulation or Oocyte Retrieval
1	14	45,X	–	–	–
2	14	46,XX/45,X[29,1]		–	–
3	15	46,X,del(X)(p11.23)		+	+
4	15	46,XX,t(X;8)(q22;q22)	+	–	–
5	16	45,X[19]/47,XXX[16]		+	+
6	17	46,X,add(X)(q22.3)		–	–
7	17	46,X,i(Xq)	+	–	–
8	18	* 1	–	–	–
9	18	45,X/46,X,psu dic(X)rea(X;X)(q22;p11)	+	–	–
10	20	* 2	–	–	–
11	20	45,X[3]/46,XX[97]	–	–	–
12	20	47,XXX	–	–	–
13	22	45,X[47]/47,XXX[3]		+	+
14	23	* 3	–	–	–
15	24	46,X,del(X)q(22)		+	+
16	25	45,X[2]/46,XX[98]		–	–
17	26	46,X,del(X)(q21)	–	+	+
18	27	45,X[34]/46,X,add(X)(q22)[16]	–	–	–
19	27	45,X[5]/47,XXX[1]/46,XX[194]		–	–
20	28	45,XX,der(14;21)(q10;q10)	–	–	–
21	28	47,XXX/46,XX	–	+	+
22	29	46,X,t(X;20)(q22;p11.2)	–	+	–
23	30	45,X/47,XXX	–	+	+
24	30	45,X[2]/46,XX[198]	+	+	+
25	30	45,X[3]/47,XXX[3]/46,XX[94]	–	–	–
26	30	46,X,del(X)(q26)[46]/45,X[3]/47,XXX[1]	–	–	–
27	30	46,XX,r(18)[23]/46,XX[27]	–	–	–
28	32	46,XX,t(7;14)(q36;q14)	–	+	–
29	32	46,XX/45,X/47,XXX	+	–	–
30	33	45,X[4]/46,XX[45]/48,XXXX[1]	–	+	–
31	33	45,X[5]/46,XX[92]/47,XXX[3]	–	+	–
32	33	46,X,del(X)(q22.3)	–	+	+
33	33	46,X,del(X)(q24)	+	–	–
34	35	45,X[2]/47,XXX[2]/46,XX[96]	–	–	–
35	35	45,X[8]/46,XX[42]		–	–
36	35	46,X,del(X)(q25)		+	+
37	36	46,X,i(X)(q10)		–	–
38	36	45,X[7]/46,XX[23]		–	–
39	37	45,X[4]/46,XX[96]	–	–	–
Robertson Translocation					
1	28	45,XX,der(14;21)(q10;q10)	–	+	+
2	29	45,XX,der(14;21)(q10;q10)	–	+	+
3	Irregular Periods	45,XX,der(13;14)(q10;q10)	–	+	+

* 1: 47,XXX [1]/47,XX,+mar1 [1]/47,XX,del (1) (q21),+mar2 [1]/46,X,del (X) (p11.2) [1]/46,XX,t (1; 10) (q23-25; p11.2-13) [1]/46,XX,t(7;14)(q36;11.2)[1]/45,XX,-20[1]/46,XX[194] * 2: 46,XX,t(3;5;15;12)(3pter→3q21::5q15→5q22::12q13→12qter;5pter→5q15::3q21→3qter;15pter→15q13::5q22→5qter; 12pter→12q13::5q15or5q22::15q13→15qter)

* 3: 46,X,add(X)(q21 or q22),add(18)(p11)[44]/45,X[6].

Robertsonian translocation occurs at the rate of 1/1,000 and is known to be associated with an increased prevalence of miscarriage, but ovarian function has been thought to be preserved. However, we had three cases of this karyotype among 688 spontaneous POI cases, suggesting a higher frequency of this karyotype in POI patients (**Table 2**) (11).

Among the women with abnormal karyotypes and secondary amenorrhea, a significant proportion of patients were older than 35 years at the onset of amenorrhea, so an age limit for testing for chromosomal abnormalities should not be set in diagnosing patients with POI (**Table 2**) (10, 11, 13).

### Genetics

Over 50 genes have been found to be involved in the etiology of POI, and many others have been implicated (14).

Most of the genetic studies in POI were conducted on genes already known to play a role in folliculogenesis (*NR5A1*, *NOBOX*, *FIGLA*, and *FOXL2*), as folliculogenesis growth factors (*inhibin A*, *GDF9*, and *BMP15*), or in ovarian steroid genesis (*FSHR*, *FSH*, *LHR*, and *LH*) (15–17). The disease course of POI differs significantly among the causative genes, the types of mutations, or possibly combinations of mutated genes that are mostly unknown at present. Despite numerous genes implicated in the etiology of POI, most women with isolated POI do not undergo genetic testing.

In clinical practice, genetic investigation is not often performed, mainly because of the cost of sequencing individual genes, and the fact that most genes account for only a small portion of POI patients. Whole exome sequencing (WES) has been used in research for a number of years and has proven useful in POI gene discovery (18).

If tests such as WES were to be utilized in a diagnostic setting, especially in infertility treatment of POI, it may enable clinicians to foresee the possibilities of fertility treatments for POI patients in the future (18).

The representative genes whose mutations are significant features in the syndromes and on family history are *FOXL2*, *CLPP*, *FSHR*, and *FMR1* (19–21). Mutations in *FOXL2* have been found to be associated with POI, in the form of BPES type 1. *FOXL2* is one of the genes responsible for the formation of the gonads and is known to be expressed in the processes that form ovarian follicles and eyelids. In humans, *FOXL2* mutations present as BPES through dominant inheritance. There are two types of BPES, both of which include malformations of the eyelids. Type I, but not Type II, is associated with POI. The ovarian phenotype of women with *FOXL2* mutations is variable. Meduri et al. reported two patients with *FOXL2* mutation; one had a follicle maturation blockage similar to that observed in the mouse model, and the other had apparently normal ovarian histology but an altered ratio of primordial to primary follicles and a tendency to develop ovarian cysts (22). Patients with BPES and presumed *FOXL2* mutation can also have streak ovaries (23). These reports indicate that *FOXL2* mutation can cause different ovarian phenotypes (22, 23).

Perrault syndrome is a disorder that causes sensorineural hearing loss, as well as ovarian failure in women. The hearing loss can range from a minor disability to a serious illness if it presents prior to the acquisition of language, whereas the ovarian dysfunction can be a serious problem that presents as primary amenorrhea. Four types of responsible genes have been identified: *C10ORF2*, *CLPP*, *HARS2*, and *LARS2*. In all four cases of POI with suspected Perrault syndrome we have encountered so far, the POI presented early, and ovulation induction was problematic. Diagnosis of Perrault syndrome requires the proper clinical setting and patient presentation. When hearing abnormalities are mild, it may be difficult for reproductive specialists to recognize. Even with the same gene mutation, the clinical presentation of Perrault syndrome may vary (24, 25).

Mutation of the *FSHR* gene has been known to cause so-called gonadotropin-resistant ovary syndrome (ROS), which is mentioned in the *Symptoms* section. There are few genes for which an association between particular mutations and their phenotypic consequences has been elucidated. *FSHR* is one of those few genes, and its mutation typically causes primary amenorrhea and impaired follicle growth, as seen in the Finnish population (26).

The fragile X mental retardation gene (*FMR1*), which contains a polymorphic CGG trinucleotide repeat in its 5` untranslated region, has been known to be associated with POI (21). The fully expanded form, which contains >200 CGG repeats, causes the loss of the RNA-binding *FMR1* protein and results in fragile X intellectual disability, mainly in men (27, 28). Premutation alleles that expand to >200 repeats over several generations have been defined in families with FMR syndrome. The premutation range is defined as being between 50 to 199 repeats, and its carriers have an increased prevalence of POI (29–31). The risk of developing POI in western women who carry the premutation is 13 to 26% (30, 31). More recently, it has been reported that, in addition to POI, the average age at menopause onset among premutation carriers is approximately 5 years younger compared to that of noncarriers (32–35). Our study of 286 sporadic, spontaneous POI patients showed six alleles in the intermediate range and two in the premutation range in five and two patients, respectively, but none were identified in normal controls. The prevalence of *FMR1* premutation in Japanese POI patients was 1.56% (2/128). The prevalence of having >36 CGG repeats in the *FMR1* gene was significantly higher in patients with POI than in normal controls, and the age at onset of amenorrhea was significantly younger in patients with >38 repeats. Thus, more than 36 CGG repeats in *FMR1* might intensify its role in the etiology of POI, at least up to the premutation range (36).

This review discusses the mutations of *AIRE*, the autoimmune-related genes, in the next section.

### Autoimmunity

POI is frequently associated with autoimmune disorders, more than in the general population, and autoimmune disorders are more frequently seen in POI patients than in the general population (37).

In our data from the St. Marianna University School of Medicine and the Rose Ladies Clinic, the total positive rate for the 16 autoantibody tests (antinuclear antibody, anti-DNA antibody, anticentromere antibody, anti-SSA antibody, anti-SSB antibody, anti-SM antibody, anti-SCL-70 antibody, anti-RNP antibody, anti-Jo-1 antibody, anti-thyroglobulin antibody, anti-cytoperoxidase antibody, microsome test, thyroid test, anti-cardiolipin antibody, anti-CL/β2GP1 antibody, and rheumatoid factor) that we tested on idiopathic, normokaryotypic POI patients was 45%. Forty-one patients (15% of patients with positive autoantibodies) were diagnosed with clinical autoimmune diseases. The autoimmune disorder most frequently associated with POI in our data was hypothyroidism, followed by hyperparathyroidism (**Table 3**).

**Table 3:** Autoimmune disorders in POI cases (St. Marianna University School of Medicine and the Rose Ladies Clinic) (11).

In 608 normal karyotype POI patients:	
Rate of patients with positive autoantibodies	44.9% (273/608)
Rate of patients diagnosed to have autoimmune disorders among the ones with positive autoantibodies	15.0% (41/273)
List of autoimmune disorders Hypothyroidism (Hashimoto’s disease): n = 20; Hyperthyroidism: n = 7; SLE: n = 4; Type I diabetes mellitus: n = 2; Addison’s disease: n = 2; Sjögren’s syndrome: n = 2; Behçet’s disease: n = 1; Dermatomyositis: n = 1; Scleroderma: n = 1; Mixed connective tissue disease (MCTD): n = 1	

The existence of ovarian autoantibodies was shown by indirect immunofluorescence on cryostat sections (38). Although autoimmunity and autoimmune disorders are frequently associated with POI, Hoek et al. reported that, histologically, oophoritis can be detected only in patients with circulatory adrenal or ovarian autoantibodies (39).

Unlike our data, Silva et al. reported that POI of adrenal origin is the most frequent type, observed in 60–80% of patients with autoimmune POI (40). Likewise, adrenocortical antibodies (ACAs) and, more specifically, 21-hydroxylase antibodies (21 OH-Abs) appear to be the markers with the highest diagnostic sensitivity for autoimmune POI. In the presence of peripheral 21 OH-Abs, SCAs were seen on cryostat sections of ovaries in over 90% of cases. In the absence of 21 OH-Abs, less than 0.5% of POI patients will be positive for SCA, a frequency not significantly different from that of the general population. Screening for 21 OH-Abs (or ACAs) should be considered in women with idiopathic POI.

Welt et al. reported the common association between POI and thyroid disease and therefore proposed that thyroid peroxidase autoantibodies (TPO-Abs) should be evaluated on a yearly basis if they are positive, and screening can be done at 5-year intervals if negative. Thus, screening for thyroid (TPO-Ab) antibodies should be performed in women with POI of unknown cause or if an immune disorder is suspected.

The association of autoimmune Addison’s disease with POI is most widely known in the context of autoimmune polyendocrine syndrome (APS). In POI patients, SCA is reportedly detectable frequently, even from before diagnosis.

APS-1 is an autosomal recessive disease caused by mutation in the *AIRE* gene, presenting mainly in childhood with Addison’s disease, candidiasis, and hypothyroidism. Ovarian insufficiency occurs in 15% of the cases (41). The syndrome includes Addison’s disease, autoimmune thyroid disease, and/or type 1 diabetes mellitus, with an array of less common organ-specific autoimmune conditions. Ovarian insufficiency occurs in approximately 10% of the cases. Because POI is one component of the autoimmune polyglandular syndromes, other autoimmune conditions may follow the diagnosis of ovarian insufficiency.

In addition to these autoantibodies, it has been reported that autoantibodies directed to different targets, including the luteinizing hormone (LH) receptor, FSH receptor, and zona pellucida are defectable in POI (42–44).

### Iatrogenic POI

Data at St. Marianna University School of Medicine and the Rose Ladies Clinic of 827 POI patients showed that 131 (15.8%) had possible iatrogenic causes (**Table 1**) (11).

**Figure 1:**
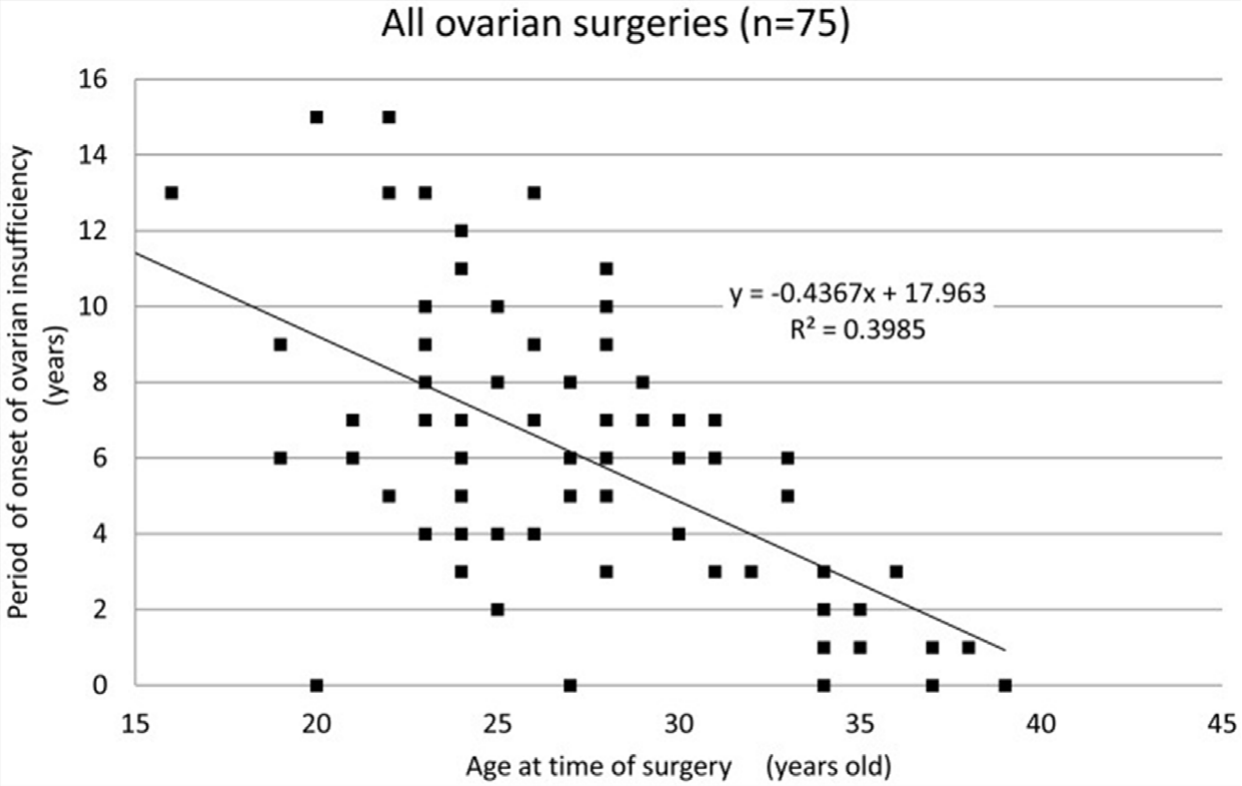
Period of onset of ovarian insufficiency post-ovarian surgery for each patient by age. A strong correlation is observed between age at the time of surgery and the period of onset of ovarian insufficiency (correlation coefficient: −0.63, Spearman’s rank correlation coefficient) (45).

Of the iatrogenic POI cases, 64% occurred following ovarian surgeries (i.e., except for bilateral oophorectomy). We reported an analysis of 75 patients who underwent surgery for benign ovarian cysts prior to the onset of ovarian insufficiency. Of these 75 patients, 66 (88.0%) underwent cystectomy. For the majority of the 75 patients, the surgical indication was the presence of endometriotic cysts (57 patients; 76.0%). Twelve patients (16.0%) underwent multiple surgeries (all bilateral cystectomies). The mean age of the patients at the time of surgery was 27.8 ± 5.5 years, and the mean period of onset of ovarian insufficiency was 5.8 ± 3.8 years. In patients with cystectomy, their age at the time of surgery and period of onset of ovarian insufficiency were well correlated (coefficient of correlation: hemilateral endometriotic cystectomy −0.64, bilateral endometriotic cystectomy −0.61, and multiple endometriotic cystectomy −0.40). We found that cystectomy of endometriotic cysts is a potential risk factor for ovarian insufficiency after surgery, with, at times, the onset of ovarian insufficiency long after cystectomy. Some patients may be unaware that their menstrual disturbance is causally related to the ovarian surgery. Therefore, it is important to monitor ovarian reserve for an extended period of time after ovarian surgery. It is particularly important to monitor ovarian reserve over the long-term in patients who wish to conceive in the future and to suggest a variety of infertility treatments appropriate for their ovarian reserve (**Figure 1**) (45).

The 5-year survival rates after cancer treatment range from 63% in general to as high as 80% for childhood cancers, and, currently, as far as young Hodgkin’s lymphoma patients are concerned, the 5-year survival rate has been improved to close to 10% (46–49). Radiotherapy and chemotherapy used to treat malignant or benign diseases can cause POI (50, 51). The risk of developing POI after radiotherapy is dependent on the radiation therapy field (abdominal pelvic radiation, total body irradiation) and on dose and age (52–54). In addition, the gonadotoxic effect of chemotherapy is largely drug and dose-dependent and related to age (55).

Alkylating agents are a typical type of gonadotoxic agent in childhood, as well as in adulthood (52, 53, 56, 57). Childhood cancer survivors treated with ovarian irradiation and alkylating agents showed a lower ovarian reserve and substantial difficulties in conceiving compared with survivors treated solely with non-alkylating agents in a 10-year follow-up study (58). Thus, the possibility of POI being the consequence of surgical and medical treatment should be discussed with women of reproductive age or younger as part of the process of obtaining informed consent for the treatment (59).

### Infectious Disease

It has been shown in case reports that viral infections can be followed by ovarian failure. However, only mumps oophoritis has been considered to be a cause of POI, accounting for 3–7% of POI cases (60). In our data of 827 POI patients at St. Marianna University School of Medicine and the Rose Ladies Clinic, eight normokaryotypic patients without possible autoimmune or iatrogenic causes had a past history of either mumps (N = 2) or appendicitis with signs of peritonitis (N = 6) in childhood (**Table 1**) (10). However, there have been only case reports, and no established data that clearly show infectious disease as a cause of POI have been reported (61).

## Symptoms and Complications

Women with POI may present with typical menopausal symptoms, sometimes even from before the onset of menstrual irregularities.

Menstrual disorders or infertility is often present several years prior to meeting the above diagnostic criteria. In cases with secondary amenorrhea, patients may experience sudden onset of amenorrhea, but amenorrhea may also be preceded by menstrual cycle changes (oligomenorrhea or polymenorrhea) (2). Although the change is fundamentally irreversible, temporary remission occurs in many cases. In a study of 358 idiopathic POI patients, spontaneous remission of ovarian function occurred in 24% of all cases, and 88% of the cases occurred within 1 year of diagnosis (62). In a more recent study of 507 idiopathic POI patients, 117 (23%) patients showed features of ovarian function resumption (63). The decrease in ovarian reserve is presumably continuous rather than stepwise, but there are no sensitive biochemical markers for evaluating the remaining follicle pool in POI patients.

Maternal age at pregnancy and age-related infertility are steadily increasing and, consequently, the demand for assisted reproductive technology (ART) is increasing. Delay of childbearing is common in developed countries, and many women are visiting infertility clinics who are past the optimal age for conception. Thus, many are presenting with a diminished ovarian response to standard ovulation induction protocols. ESHRE published the Bologna criteria for poor ovarian response (POR) in 2011, which included antral follicle count (AFC) less than five to seven follicles and anti-Mullerian hormone (AMH) levels below 0.5–1.0 ng/ml, as well as a previous history of a poor response (≤3 with conventional stimulation protocols) and advanced age (>40 years). POR may include several sub-populations and may represent the early stage of POI. Therefore, if an infertile patient presents in the above category, especially when the patient is younger than 40 years of age, POI should be ruled out.

ROS has been described as a specific entity of primary or secondary amenorrhea with normal development of secondary sex characteristics in association with hypergonadotropinism and the presence of normal or subnormal ovarian follicle reserve, as could be seen in POI patients with *FOXL2* mutations (22, 64, 65). Histological examination and ultrasound scanning show primary, secondary, preantral, and even antral follicles. It is also considered to be one step towards established POI, but there are also certain cases of FSH receptor mutation that persistently show the typical pattern of ROS (65).

### Vasomotor Symptoms and Psychological Impact

**Figure 2:**
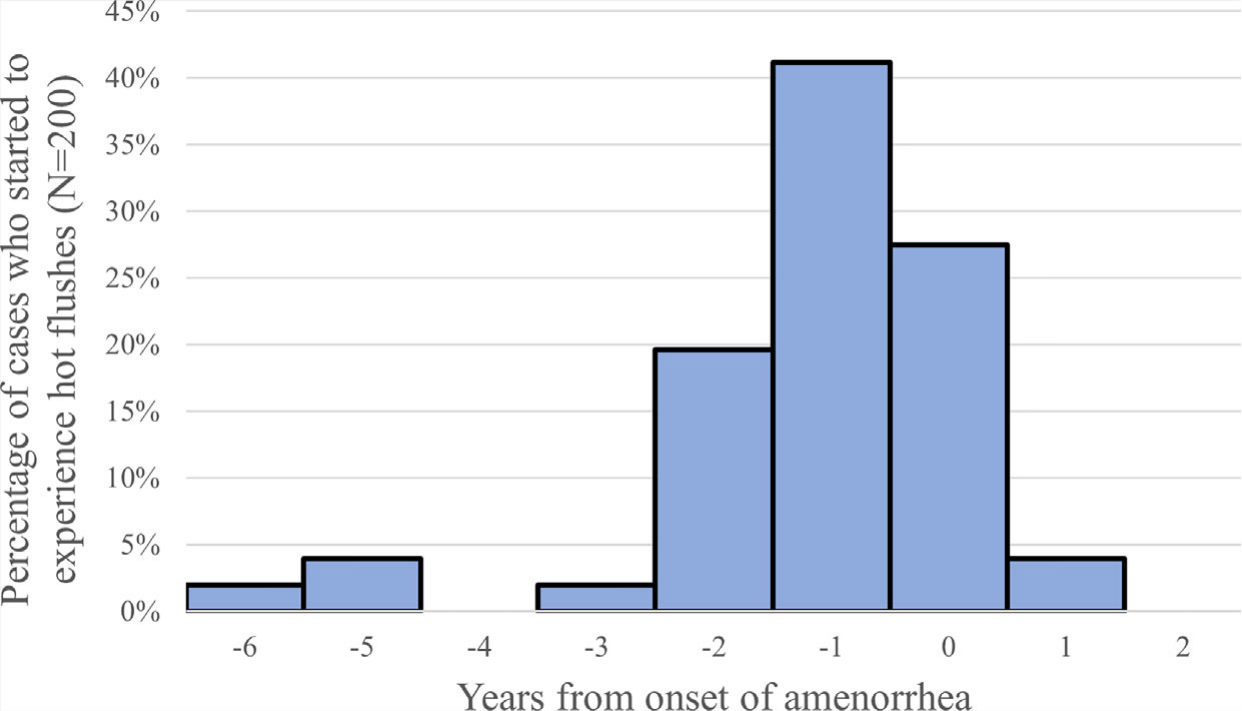
Timing of onset of hot flushes (year from the onset of amenorrhea), 66% (132/200) of the POI patients reported the experience of hot flushes (11).

Sixty-six percent of the POI patients at the St. Marianna University School of Medicine experienced hot flushes, and most of them were between 2 years prior to and the year of onset of amenorrhea (**Figure 2**) (11). Although age is not a factor in the prevalence of hot flushes among women 25 years old or older, those under 25 years of age experience them less frequently. Women who undergo surgical menopause experience severe and persistent symptoms. These observations suggest that these symptoms are age-related and caused by estrogen withdrawal and not by estrogen deficiency.

Vasomotor symptoms are associated with sleep problems, which may deteriorate mood states, social participation, and work performance, as well as overall health-related quality of life (66). The cross-sectional community survey of menopausal symptoms among Japanese perimenopausal women conducted in Kawasaki city showed that 49 and 45% of them experienced hot flushes and insomnia, respectively. In addition, depression, headache, and fatigability were experienced by 50, 38, and 63%, respectively (67). A POI diagnosis itself has considerable effects on a patient. Many women experience depression and/or reduced libido due to the sense of loss of reproductive function and physical changes, such as vaginal dryness (68, 69).

### Neuro-Psychological Symptoms

Declining short-term memory and cognitive function and an increased incidence of Alzheimer’s disease have been reported in patients with POI, but these phenomena have not been observed before or after the age-appropriate menopause (70–72). Oophorectomy before the age of menopause increases the risk of cognitive impairment or dementia nearly two-fold (73–77). These data suggest that early estrogen deficiency has an effect on cognitive function. However, the data are primarily derived from post-oophorectomy patients.

### Urogenital Symptoms

Estrogen deficiency leads to urogenital atrophy, causing common urogenital symptoms such as vaginal dryness, vaginal irritation, and itching (78). The symptoms have been widely studied in women undergoing an age-appropriate menopausal transition, but there are few studies concerning the prevalence and treatment of urogenital symptoms in POI patients.

### Cardiovascular Disease

Patients with POI have been reported to have shortened life expectancy (1, 79). The main reason for this is considered to be cardiovascular disease. Women with POI have been reported to have several risk factors for the development of cardiovascular disease: endothelial dysfunction, autonomic dysfunction, abnormal lipid profile, insulin action disturbances, and metabolic syndrome (80).

Flow-mediated dilation of the brachial artery, as an indication of endothelial function, has reportedly been found to be reduced significantly in POI patients. In addition, the number of circulating endothelial progenitor cells is decreased in correlation with a decrease in serum estrogen levels (81, 82). POI patients have an increased carotid intima media thickness and left ventricular diastolic function (83). Moreover, reduced heart rate variability and impaired baroreflex sensitivity compared to healthy controls have been shown in POI patients (84).

POI patients show abnormalities in lipid profiles, but the results are conflicting regarding particular lipoproteins. As Knauff et al. reported, patients with POI have significantly higher triglyceride (TG) levels and lower high-density lipoprotein cholesterol (HDL-C) levels compared to controls, whereas Ates et al. reported increased total cholesterol (TC) and HDL cholesterol in POI women compared to healthy controls (83, 85). The analyzed population showed similar levels of glucose, insulin, homeostasis model of assessment-insulin resistance (HOMA-IR), low-density lipoprotein cholesterol (LDL-C), and triglycerides as the controls, but the incidence of metabolic syndrome was significantly increased (80, 85). In contrast, other authors detected increased serum glucose, insulin, and HOMA-IR in POI women *vs.* controls (80, 86).

The overall cardiovascular risk in POI women appears to be significantly increased despite the conflicting data regarding the lipid profile and insulin resistance indices (80). In particular, the risk of mortality from ischemic heart disease is increased approximately 80% in POI women compared to women with menopause at 49–55 years (80, 87).

### Bone Mineral Density

It has been clearly established that estrogen deficiency in postmenopausal women is associated with osteoporosis (North American Menopause Society) (88). Albright et al. first reported the relationship between an increased incidence of fractures and postmenopausal estrogen deficiency (89, 90).

In young women, hypoestrogenism and hypoandrogenemia affect peak bone mass (PBM) formation and bone mineral density (BMD) status (91). Lana et al. reported that serum FSH concentrations, but not estradiol concentrations, are positively associated with bone mass loss in skeletal regions (both the spinal column and femoral neck) in patients with spontaneous POI (80, 90).

Numerous studies have reported a significant decrease of BMD in POI patients (7, 92–95). Uygur et al. found that both spinal bone BMD and femoral neck bone BMD were significantly lower in POI patients than in controls (96). A study of 442 cases confirmed that POI patients have a lower BMD compared to regularly menstruating women (93). A study by Nelson et al. reported that 67% of patients with POI have osteopenia (97). Leite-Silva et al. studied 50 women with POI and found a decrease in femoral and lumbar spine BMD (98). The lumbar part of the spine was the most affected by the BMD decrease. They reported that age generally, age at the onset of POI, and reproductive age were factors associated with BMD of the lumbar spine. Total body BMD is significantly correlated with the duration of ovarian function in POI patients (80, 99).

There are limited data regarding fracture risk in POI patients. Clinical studies that compared women experiencing menopause at a normal age to women who had premature menopause reported a relative risk for fracture of approximately 1.5 in women with premature menopause (80, 100).

### Type 2 Diabetes

There is conflicting data on changes in insulin resistance indices in POI patients, and the association between age at menopause and the risk of developing type 2 diabetes mellitus (DM) has not been well established (80, 86). Recently, a systemic review and meta-analysis by Anagnostis et al. reported that patients with POI have a higher risk of developing type 2 DM as compared to women with normal menopause (OR: 1.53, 95% CI:1.03–2.27, p = 0.035) (101). Although the mechanisms underlying the association between POI and type 2 DM have not been fully elucidated, it is possible that shorter exposure to endogenous estrogens play a role in the pathogenesis because of their protective effect on pancreatic β-cell function and insulin resistance (101). Estradiol, through binding to its alpha receptor (ERα) in β-cells and through the concomitant phosphorylation of extracellular signal-regulated kinases (ERK1/2), regulates insulin biosynthesis and secretion and modulates β-cell survival. Thus, women with POI are at a high risk for developing type 2 DM, especially when other risk factors, such as positive family history for type 2 DM or obesity co-exist. In these women, an earlier life-style intervention compared with the general population should be advised (101).

## Treatment

With all of the above-mentioned physical and psychological conditions, treatment should focus on the maintenance of the patient’s well-being. Infertility treatment used to be thought of as being of no or little value. Recently, some attempts to improve fertility in POI patients have been reported. In the following section, this review attempts to summarize the recent advances in augmenting the well-being of POI patients, as well as trials in the field of fertility in POI patients.

### Hormone Replacement Therapy (HRT)

HRT must be performed when the diagnosis of POI is made, except where contraindicated.

As mentioned previously, estrogen deficiency in POI patients is known to cause the early aging of blood vessels, bones, and other tissue and shorten patients’ life expectancy (102, 103).

The occurrence of vasomotor symptoms seems to be a major reason for POI patients to start receiving hormone replacement. Data on women with iatrogenic POI support the effect of HRT in relieving vasomotor symptoms (104). Of the POI patients who had undergone chemotherapy, 66% of patients who were on HRT reported a significant reduction in hot flushes, insomnia, and psychological and emotional changes (105). Urogenital symptoms of vaginal dryness, irritation, urinary frequency, and incontinence were also less prevalent in women with POI using HRT compared to non-users (105–108).

There is evidence that HRT reduces the impact of POI on bone health (109–113). Large, randomized trials have shown that HRT in postmenopausal women improves BMD and reduces hip and vertebral fracture risk (114–116).

Estrogen replacement has been demonstrated to have beneficial effects on BMD in women following premenopausal oophorectomy. In a placebo-controlled study of 58 women (mean age 48 years, followed for an average of 9 years) after oophorectomy, mestranol reduced bone loss, with less reduction in vertebral body height (117). Similarly, in 33 women (mean age 45 years) taking conjugated equine estrogen (0.625 mg, with calcium supplementation) for 1 year after oophorectomy, spine BMD did not decrease significantly (−1.5%), whereas it decreased by 6.1% in women taking medroxyprogesterone acetate. Estrogen treatment also decreased the elevation of bone resorption markers following oophorectomy (109). With respect to cardiovascular health, HRT of 6 months’ duration improved flow-mediated dilation of the brachial artery by 2.4-fold, similar to the levels in healthy controls (118). Goldemeier et al. have also reported normal endothelial-dependent vasodilation in POI women on HRT (118). Some studies have shown that combined HRT (i.e. estrogen and progesterone) restored endothelial dysfunction in women with POI, decreased the risk of ischemic heart disease, and prevented the increase in cardiovascular disease mortality associated with bilateral oophorectomy (82, 102, 103, 119, 120).

Thus, HRT is indicated for the treatment of vasomotor and urogenital symptoms in women with POI. It is also recommended to maintain bone health and prevent osteoporosis. HRT also appears to have a role in the primary prevention of cardiovascular disease.

Estrogen replacement mitigated the decline in cognition associated with early oophorectomy when patients were treated within 5 years of surgery and for a duration of at least 10 years (73, 74, 103). In contrast, studies of oophorectomy before the natural menopause and the risk of dementia have shown conflicting results, which may be attributable to the difference in timing of the initiation of HRT (121, 122). It has been suggested that HRT may have neuroprotective effects when provided close to the menopausal transition, whereas HRT may have detrimental effects and may increase the risk of cognitive impairment if given to older women. These adverse effects in older women may be related to existing vascular or neurological disease, or to an increased risk of venous thromboembolism (VTE). Considering the long duration of hormone replacement in POI patients, transdermal 17β-estradiol is preferable. Oral contraceptives that have a pharmacological rather than a physiological replacement dose have adverse effects on the lipid profile and hemostatic factors, plus an increased risk of (VTE) (76, 103, 123–125). These results suggest that HRT should be initiated at the earliest possible time after the diagnosis is made.

There is little evidence of the effects of various progestins in HRT for women with POI. Evidence from older physiologically postmenopausal women favors micronized natural progesterone, because it is associated with a better cardiovascular profile and possibly reduced breast cancer risk, with similar efficacy for protecting the endometrium (114, 126, 127). Continuous estrogen replacement is desirable to avoid symptoms of estrogen deficiency. Some women using oral contraceptives will be symptomatic during the pill-free period, and the conventional 3 + 1 regimen results in no hormone replacement for 25% of the time. Cyclical rather than continuous, combined regimens stimulating active functioning of the endometrium are necessary for women desiring pregnancy by oocyte donation (128). This may lead to a slightly higher risk of hyperplasia/carcinoma of the endometrium (129, 130). Cycle length can be individualized, but probably should not exceed 12 weeks to protect the endometrium. Continuous, combined HRT decreases the risk of endometrial cancer in postmenopausal women and probably for women with POI (129). Progestins can be administered orally or transdermally. No studies that compared the route of administration of progesterones for POI patients were identified. However, as in normal postmenopausal women, it is assumed that the effectiveness of endometrial protection would be similar between young and old menopausal women. If women prefer a bleed-free regimen, a progestogen-releasing intra-uterine system provides sufficient protection from endometrial hyperplasia (131). In conclusion, women with POI should be informed that HRT before the age of normal menopause is mandatory if not contraindicated. It has not been found to increase the risk of breast cancer. Progestogen should be given in combination with estrogen to protect the endometrium, except for women after hysterectomy. POI patients with lifestyle risk factors for VTE should be advised to reduce them.

### Infertility Treatment

Due to the increasing trend of late marriage and/or childbearing in developed countries, the cumulative incidence of POI has increased among women who wish to conceive. POI is becoming an important clinical issue requiring infertility treatment. In a study of 358 idiopathic POI patients, spontaneous remission of ovarian function indicated by the resumption of menstrual cycles and/or a decrease in FSH levels to the normal range occurred in 24% of all cases. The spontaneous pregnancy rate in these patients after diagnosis was 4.4% (62). Bachelot et al. also reported in their cross-sectional study of 507 idiopathic POI patients that 117 (23%) of the cohort experienced spontaneous resumption of ovarian function, and 18 (3.6%) conceived spontaneously (63). Furthermore, in observational studies of POI patients who had received estrogen replacement, the pregnancy rate was 4.8% (132). Attempts at ovulation induction in infertile POI patients yielded an overall pregnancy rate of 6.3%, and controlled studies using gonadotropin-releasing hormone agonist (GnRH-a) suppression of gonadotropins *versus* placebo failed to show any difference in pregnancy rates (132–134). Thus, oocyte donation (OD) was considered to be the most reasonable treatment option for infertility in POI patients (132, 135).

The lack of ability to have their own genetic offspring remains a significant concern for women with POI. Furthermore, in certain cultures, egg donation is prohibited. These women are actively seeking and strongly requesting any available treatments that could improve their chances of pregnancy and taking home a baby.

More recently, Tartagni et al. and Badawy et al. reported randomized studies of ovulation induction in POI patients (136, 137). Tartagni et al. performed a randomized trial involving 50 women with POI treated with ethinylestradiol (EE) or placebo 2 weeks before and during gonadotropin treatment, with the main outcome being ovulation (137). Eight of 25 women treated with EE ovulated, and four of them conceived. None of the 25 women in the placebo group ovulated. Ovulation occurred in women with FSH levels <15 mIU/ml during EE treatment. In the study by Badawy et al., 58 idiopathic POI patients with GnRH-a and gonadotropin therapy were randomized to receive additional dexamethasone or placebo, assuming that some of the patients may have an autoimmune etiology of POI (136). Ovulation was detected in six of 29 women treated with dexamethasone *versus* three of 29 in the placebo group. This difference was significant, but only a cautious conclusion can be drawn due to the small size of the study. These data, according to the ESHRE POI Guideline Development Group, confirm the high rate of follicle development and potentially of ovulation in women with POI, especially with a shorter duration of amenorrhea. The potential beneficial effect of immunosuppression in POI of possible autoimmune etiology has been reported only on a case report basis (138, 139).

There has been an overall increase in post cancer treatment POI patients followed by an increase in long-term survival of the affected patients. Protection against iatrogenic POI caused by chemotherapy, radiation therapy, or surgery assumes a high priority. Shielding or ovarian transposition during radiotherapy and fertility-sparing surgery should be considered in young females undergoing cancer treatment. GnRH analogue administration during chemotherapy significantly decreased the risk of POI in young cancer patients, but it did not exhibit protective effects for fertility (140, 141). Cryopreservation of embryos and mature oocytes is the clinically established method, with pregnancy rates and live births reaching 25% (142, 143). Novel methods such as retrieving immature oocytes aiming at maturing them later *in vitro* and freezing of gonadal tissue are very promising, but still considered experimental (144).

Among previous uncontrolled interventional studies, most pregnancies were obtained in a study in which they attempted to suppress gonadotropins with GnRH-a and estrogen replacement followed by human menopausal gonadotropin (hMG) stimulation (145). However, few past studies attempted long-term ovulation induction by hMG/recombinant FSH (recFSH) with gonadotropin suppression by GnRH-a under estrogen replacement over cycles. Because it takes several months for follicle maturation from secondary-preantral to the preovulatory stage, longer stimulation duration with hMG/recFSH under estrogen replacement and GnRH-a treatment may be more effective to induce follicle growth in established POI patients (146). In this regard, ovulation induction by long-term ovarian stimulation with high-dose hMG/recFSH, with gonadotropin suppression with estrogen replacement with GnRH-a, especially by suppressing serum LH levels in POI patients, may increase ovulation and pregnancy rates.

It was recently suggested that women with POI and an abnormal karyotype might have an inferior chance of conceiving from their own eggs compared to POI from non-genetic causes (147). However, the literature concerning the reproductive outcomes of POI with an abnormal karyotype is still scarce.

Platelet-rich plasma (PRP), a plasma fraction of autologous blood with a high concentration of platelets (148), has been used in regenerative medicine in the last decade (149–152). The plasma contains a broad spectrum of growth factors that have been suggested to be able to enhance angiogenesis regeneration and the cell proliferation process (153). In the ovary, these growth factors are known to play important roles in modulating folliculogenesis (154–156). Many case reports and case series showed some good outcomes in infertile women with a poor prognosis for fertility, including POI patients and poor responders with POR (157–161).

Recently, a method for activation of dormant follicles using *in vitro* culture of ovarian fragments treated with PI3K stimulators and *PTEN* inhibitors in humans has been developed (162). Subsequent studies suggested that ovarian fragmentation itself could interfere with the ovarian Hippo signaling pathway, leading to ovarian follicle growth (163). Kawamura et al. combined these two methods in an *in vitro* activation (IVA) approach to treat infertility in patients with POI (164). Two successful full-term births were reported after IVA in established POI patients (165).

Because patients with POR or POI in the early stage supposedly have spontaneous activation of dormant primordial follicles to the secondary stage, secondary follicle growth could be promoted using fragmentation and immediate re-implantation of the ovarian cortex tissue without tissue culture (drug-free IVA). We have reported that nine of 11 POR patients showed increased antral follicle numbers in multiple growth waves detected following drug-free IVA and hMG/FSH treatment. Thus, these approaches may be suitable for POR or POI in the early stage (166).

These new approaches require further modification and evaluation by randomized, controlled trials. However, they may open new prospects for the treatment of infertility in POI.

## Conclusions

The etiology of POI is basically genetic, including chromosomal abnormalities, overlapped by autoimmunity, which is also partly related to genetic causes. In most of the idiopathic cases, the genetic background is unknown.

Initially, it was reported that its prevalence was 1%, but some new studies reported 1.8% or higher. Its regional and ethnic differences, which may be related to genetic differences, are still largely unclear.

The primary treatment modality is HRT, and, for POI patients, HRT is generally safe and beneficial for alleviating vasomotor and urogenital symptoms and preventing cardiovascular diseases and osteoporosis, thus improving patients’ quality of life. However, the optimal dosage, hormone preparations used, and duration of HRT for POI patients have not been fully investigated.

Due to the tendency for late childbearing, infertility caused by POI along with the age-related decrease in ovarian reserve is a serious problem in all developed countries. A trial of a new approach to fertility treatment for POI patients was recently reported (166). These attempts are important because they can be generalized to the infertility treatment of age-related diminished ovarian reserve.

In that context, a better understanding of the genetic causes of POI and development of more sensitive markers of secondary/preantral follicles are essential.

## Author Contributions

The author conceived of this review and the critical appraisal of the literature summarized herein. All authors contributed to the article and approved the submitted version.

## Conflict of Interest

The author declares that the research was conducted in the absence of any commercial or financial relationships that could be construed as a potential conflict of interest.
